# Is *Plasmodium vivax* Malaria a Severe Malaria?: A Systematic Review and Meta-Analysis

**DOI:** 10.1371/journal.pntd.0003071

**Published:** 2014-08-14

**Authors:** Cho Naing, Maxine A. Whittaker, Victor Nyunt Wai, Joon Wah Mak

**Affiliations:** 1 School of Postgraduate Studies, International Medical University, Kuala Lumpur, Malaysia; 2 School of Population Health, University of Queensland, Brisbane, Australia; 3 School of Medicine, International Medical University, Kuala Lumpur, Malaysia; Barcelona Centre for International Health Research (CRESIB) and Institució Catalana de Recerca i Estudis Avançats (ICREA), Spain

## Abstract

**Background:**

*Plasmodium vivax* is one of the major species of malaria infecting humans. Although emphasis on *P. falciparum* is appropriate, the burden of vivax malaria should be given due attention. This study aimed to synthesize the evidence on severe malaria in *P. vivax* infection compared with that in *P. falciparum* infection.

**Methods/Principal Findings:**

We searched relevant studies in electronic databases. The main outcomes required for inclusion in the review were mortality, severe malaria (SM) and severe anaemia (SA). The methodological quality of the included studies was assessed using the Newcastle-Ottawa Scale. Overall, 26 studies were included. The main meta-analysis was restricted to the high quality studies. Eight studies (n = 27490) compared the incidence of SM between *P. vivax* infection and *P. falciparum* mono-infection; a comparable incidence was found in infants (OR: 0.45, 95% CI:0.04–5.68, *I* ^2^:98%), under 5 year age group (OR: 2.06, 95% CI: 0.83–5.1, *I* ^2^:83%), the 5–15 year-age group (OR: 0.6, 95% CI: 0.31–1.16, *I* ^2^:81%) and adults (OR: 0.83, 95% CI: 0.67–1.03, *I* ^2^:25%). Six studies reported the incidences of SA in *P. vivax* infection and *P. falciparum* mono-infection; a comparable incidence of SA was found among infants (OR: 3.47, 95%:0.64–18.94, *I* ^2^: 92%), the 5–15 year-age group (OR:0.71, 95% CI: 0.06–8.57, *I* ^2^:82%). This was significantly lower in adults (OR:0.75, 95% CI: 0.62–0.92, *I* ^2^:0%). Five studies (n = 71079) compared the mortality rate between vivax malaria and falciparum malaria. A lower rate of mortality was found in infants with vivax malaria (OR:0.61, 95% CI:0.5–0.76, *I* ^2^:0%), while this was comparable in the 5–15 year- age group (OR: 0.43, 95% CI:0.06–2.91, *I* ^2^:84%) and the children of unspecified-age group (OR: 0.77, 95% CI:0.59–1.01, *I* ^2^:0%).

**Conclusion:**

Overall, the present analysis identified that the incidence of SM in patients infected with *P. vivax* was considerable, indicating that *P. vivax* is a major cause of SM. Awareness of the clinical manifestations of vivax malaria should prompt early detection. Subsequent treatment and monitoring of complications can be life-saving.

## Author Summary

Until recently, vivax malaria has received less attention than falciparum malaria and was deemed neglected. There has been a surge in studies that documented the contribution of *Plasmodium vivax* to severe malaria in some endemic countries such as Thailand, Brazil, Indonesia, Papua New Guinea and India. We aimed to synthesize the evidence on severe malaria in *P. vivax* infection compared with that in *P. falciparum* infection. We searched relevant studies in electronic databases. The combined results of the eight relatively high quality studies showed a comparable incidence between vivax malaria and falciparum malaria in infants, under 5 year age group, the 5–15 year age group and adults. The combined results of the six relatively high quality studies revealed a comparable incidence of severe anaemia between *P. vivax* infection and *P. falciparum* mono-infection in both the infants and the 5–15 year age group. Considering that severe malaria and deaths attributable to *P. vivax* is not a rare event, raised clinical awareness of the manifestations of vivax malaria in patients of any age should prompt early detection of malaria. It is likely that early detection, appropriate treatment commenced in a timely manner and close monitoring of any complications could be life-saving and contribute to the attainment of the global vision of no malaria deaths.

## Introduction

*Plasmodium falciparum* and *P. vivax* are the two major species of malaria infecting humans. Although emphasis on *P. falciparum* is appropriate, the burden of vivax malaria should be given due attention as almost 40% of the world population are at risk of vivax malaria [1]. Historical evidence from the neurosyphilis therapies in the early 1900s showed 5–15% fatality rates in the American and European treatment facilities using *P. vivax*, indicating that *P. vivax* is dangerous and not benign [2]. *P. vivax* malaria is prevalent in many regions of the world, and in Asia and Latin America it accounts for more than half of all the malaria cases [3], [4]. In 2009, it was reported that 2.85 billion people were at risk of *P. vivax* transmission and 91% of these occurred in Central and South East Asia [5].

Recently, there has been a surge in studies that reported the contribution of *P. vivax* to severe malaria (SM) in countries such as Thailand [6], Brazil [7], Indonesia [8], Papua New Guinea (PNG) [9] and India [10]. It has been well documented that severe anaemia (SA), a presenting clinical manifestation of SM, is an important determinant of infant mortality [11] in endemic areas. The incidence of clinical manifestations of severe vivax malaria has been reported from endemic areas, albeit with variations in the presenting clinical patterns. Individual studies assessing the prevalence of SM related to vivax malaria are available, but their findings are inconclusive. Reviews addressing studies in the Brazilian context only [12] and a review on SA alone [11] were available. To our knowledge, no statistical pooling of results has been undertaken. This gap in evidence stimulated us to conduct a systematic review and meta-analysis. Meta-analysis is the process of combining study results that can be used to draw conclusions. The final product has both quantitative and qualitative elements, as it takes into account the numerical results and sample sizes of the individual studies as well as the more subjective issues such as quality, extent of bias, and strength of the study design [13]. Therefore, the objective of the present study was to synthesize evidence on SM in *P. vivax* infection compared with that in *P. falciparum* infection.

## Methods

The present study followed the preferred reporting items for systematic reviews and meta-analyses (PRISMA) statements [14] (Checklist S1).

### Study search

We searched studies on the relative distribution of severe malaria in a *P. vivax* infection in electronic databases such as PubMed, Ovid and Google Scholar. The search was limited to studies published in English until February 2014. We used the following medical subject heading (MeSH) and/or text words in any field, “(vivax malaria OR vivax)” combined with “(severe malaria OR complicated malaria OR severe anaemia OR severe malaria anaemia).” The search strategy was slightly modified according to the requirements of different databases. We also looked at the references of retrieved articles and relevant reviews for any additional studies.

### Study selection

Studies were selected for the present meta-analysis if they met the following criteria:

#### Study population

Participants residing in malaria endemic countries presenting with clinical manifestations of *P. vivax* infection, regardless of age and gender. Microbiological diagnosis of malaria was made based on microscopy of Giemsa-stained blood films or a rapid-onsite diagnostic test and with species confirmation through PCR-based analysis.

#### Study design

Observational designs, prospective cohort and case-control designs (case-control/nested-case control studies carried out with a clear description of the selection of controls) were considered. Studies which compared SM between *P. vivax* and *P. falciparum* (either mono-infection or mixed infection), and/or compared patients with SM and non-SM in *P. vivax* malaria were included.

#### Study outcomes

The outcomes were SM, SA, and other common clinical manifestations related to vivax malaria. SM for *P. vivax* was defined according to standard criteria of the World Health Organization (WHO) for *P. falciparum* [15] with the exception of the criterion for parasite density. This is because *P. vivax* preferential invasion of younger red blood cells (reticulocytes) necessarily lowers the threshold at which it would be considered to be hyperparasitaemic. SA was defined as having haemoglobin levels of <7 g/dL in adults and <5 g/dL in children, according to the WHO criteria [15], [16]. For the outcome assessment, studies which provided relative risk (RR) or odds ratio (OR) estimates with its 95% confidence interval (95% CI) (or data to calculate the estimates) were included. If more than one study presented data from the same study participants, either the study of the higher quality or the most comprehensive was included.

Studies were excluded from the meta-analysis, if they (i) were case reports, (ii) assessed particular populations (e.g. non-immune travelers, pregnant mothers, patients with known co-morbid conditions), (iii) had a sample size less than 10, (iv) had measured outcomes which were not clearly presented, or (v) were studies from which appropriate data could not be extracted.

### Data extraction

Two authors independently screened the titles and abstracts of publications according to the inclusion criteria. These two authors then independently extracted information from each of the included studies using the pre-tested data extraction form prepared for this study. Information collected was author, year of publication, country, patient characteristics, sample size, mean age, gender, study design and the reported clinical outcomes. Disagreements between the two authors were resolved by consensus. The methodological quality of the included studies was assessed using the Newcastle-Ottawa Scale (NOS) [17]. The instrument used a star system to assess the study quality based on three criteria; (i) participants' selection (4 stars), (ii) comparability of study groups (2 stars) and (iii) assessment of exposure (3 stars). Hence, the highest total score for a study was nine.

### Data analyses

We assumed that the RR from cohort studies approximates OR from case-control studies [18]. In order to assess the differences between the various clinical manifestations of *P. vivax* and *P. falciparum* infections (either mono or mixed), we extracted adjusted OR and corresponding 95% CI from each study, wherever possible. If RR or OR was not reported, we calculated these from the raw data provided in the study. As the incidence of SM is not very high, a more conservative estimate of the OR was used for the pooled analysis. The heterogeneity between these studies was assessed with the *I* ^2^ test. A calculated value of *I* ^2^ over 50% indicated substantial heterogeneity. For pooling of the results, we used a more conservative random-effect model [19]. This is because even if the *I^2^* statistic is low or zero, heterogeneity could still be a concern since it is likely to be present but undetected [20]. The measure of effect sizes is the OR, but statistical analysis was carried out with its natural logarithm (i.e the log OR) since a sampling distribution is more closely approximated by a normal distribution [21]. Moreover, we thought that some of the included studies might have been poorly conducted or poorly reported. This makes the reliability of their input data questionable. Therefore, we performed meta-analyses restricted to the high quality studies (i.e. studies with <7 score). The remaining relatively low quality studies were retained for a sensitivity analysis.

If data allowed, we stratified the incidence of SM by age groups (infants, under 5 years, 5–15 years), by transmission intensity (with or without marked seasonality), by species (*P. vivax*, *P. falciparum* and mixed infections) and by chloroquine (CQ) resistance status. The presence of publication bias was assessed by visualizing funnel plots. Data entry and analyses were performed using RevMan (version 5.3) (The Cochrane Collaboration, Nordic Cochrane Centre, Copenhagen, Denmark). A protocol of this study is available [22].

## Results

**Figure 1:**
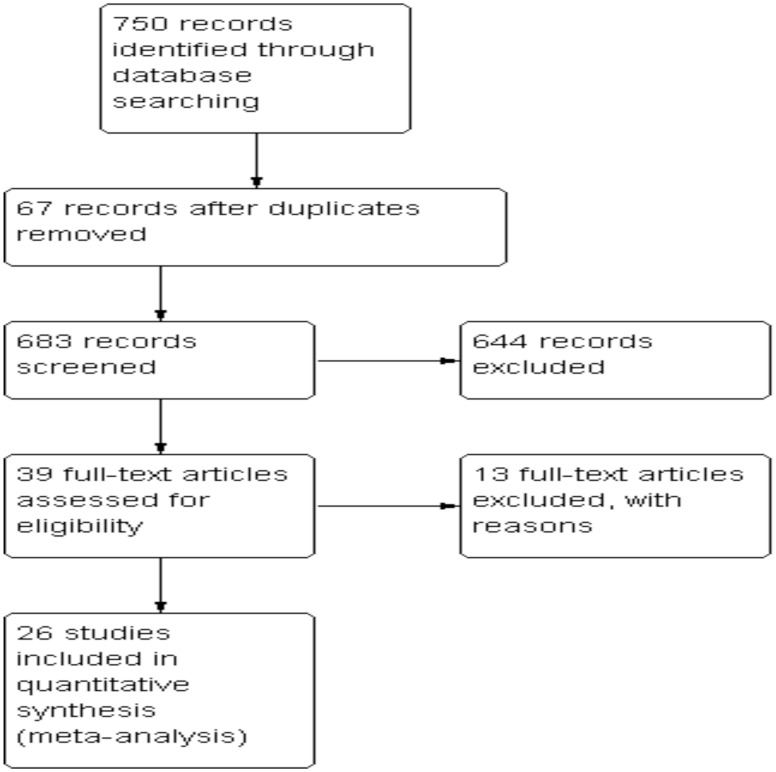
Flow diagram indicating the study selection.

Figure 1 presents the study selection process. The initial search yielded 750 citations, of which 39 potentially met the inclusion criteria. Of them, 26 studies were identified for consideration in quantitative synthesis [8], [10], [23]–[46]. Thirteen studies were excluded because (i) there was no comparator group [3], [47]–[50], (ii) there were no data on SM cases [51]–[54], (iii) there were no data on *P.vivax* infection [55], (iv) they were review articles [7], [56] and (v) they were studies of a particular population (i.e military group) [57].

### Baseline characteristics of the included studies

Table S1 presents the characteristics of the included studies. Many studies were conducted in India (n = 10) [10], [28], [31], [32], [34], [35], [37], [42], [44], [45], Indonesia (n = 5) [8], [26], [27], [40], [41]. The remaining studies came from Pakistan (n = 3) [30], [43], [46], PNG (n = 3) [29], [38], [39], Brazil (n = 2) [24], [36], Ethiopia (n = 1) [33], Malaysia (n = 1) [25] and Sudan (n = 1) [23]. Ten studies (38.4%) were published in the year 2013. Also, 38.4% of the included studies confirmed vivax malaria by PCR. Of the 26 included studies, only 10 studies (38.4%) were high quality studies (≥7 scores), on the basis of the NOS checklist in which the maximum score is 9 (Table S2). We only considered high quality studies for the main meta-analyses. The remaining studies with lower quality scores were retained for sensitivity analysis.

Some studies were low in their quality because the ‘follow-up period’ was not long enough (or failed to report this duration) for outcomes to have occurred [10], [23], [28], [30], [37], [41], [42], [44]. Some studies failed to address the assessment of ‘common outcomes’ [10], [28], [30], [35], [37], [41], [42], [44], [45]. Failure to address any of these important criteria could affect the validity of their study process and/or outcome measurements.

### Mortality attributable to vivax malaria

**Figure 2:**
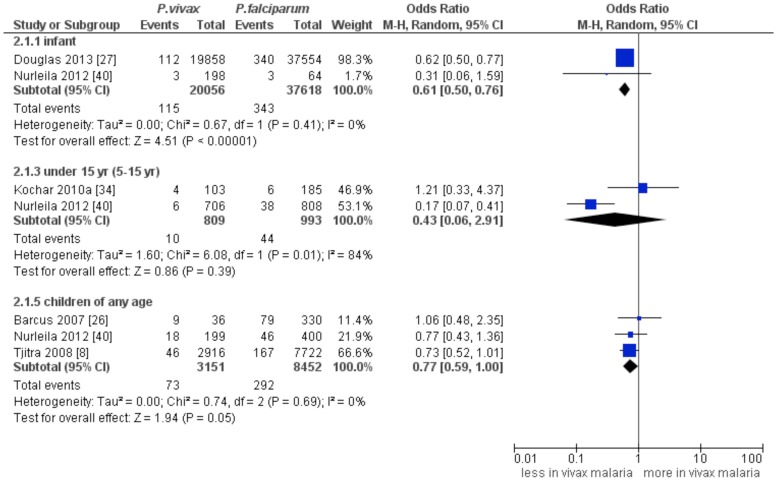
Forest plot showing a comparison of mortality between *P. vivax* and *P. falciparum* mono-infections.

Of the 26 included studies, 8 studies (n = 74079) [8], [10], [26], [27], [34], [37], [40], [43], [46] reported the percentage of mortality attributable to *P. vivax*. Compared with *P. falciparum* malaria, the mortality rate among infants was significantly lower in *P. vivax* infection (summary OR: 0.61, 95% CI: 0.5–0.76, *I* ^2^: 0%). But, the mortality rate between vivax and falciparum malaria was comparable in under 5 year-age –group (summary OR: 0.43, 95% CI: 0.06–2.91, *I* ^2^: 84%) (Figure 2).

Five studies (n = 33824) [8], [26], [27], [34], [40] reported the percentage of mortality, comparing *P. vivax* infection with mixed infection (*P. falciparum* and *P. vivax*); the mortality rates among infants (summary OR: 0.79, 95% CI: 0.58–1.08, *I* ^2^: 0%) and among children in the 5–15 year-age group (summary OR: 0.66, 95% CI: 0.12–3.74, *I* ^2^: 0%) were comparable (Figure S1).

### Severe malaria

**Figure 3:**
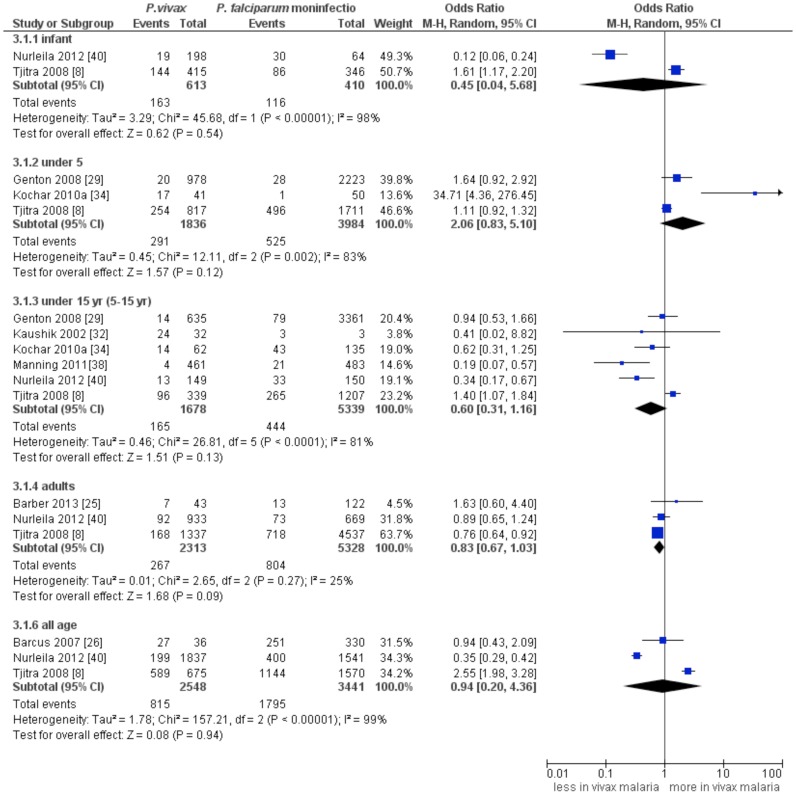
Forest plot showing a comparison of severe malaria between *P. vivax* and *P. falciparum* mono-infections.

Eight studies (n = 27490) [8], [25], [26], [29], [32], [34], [38], [40] were included in the pooled analysis. A comparable incidence of SM between *P. vivax* infection and *P. falciparum* mono-infection was found in infants (summary OR: 0.45, 95% CI: 0.04–5.68, *I* ^2^:98%), under 5 year-age group (summary OR: 2.06, 95% CI: 0.93–5.1, *I* ^2^:83%), the 5–15 year-age group (summary OR: 0.61, 95% CI: 0.31–1.16, *I* ^2^:81%), children of any age (summary OR: 0.94, 95% CI: 0.2–4.36, *I* ^2^:99%) as well as in adults (summary OR: 0.83, 95% CI: 0.67–1.03, *I* ^2^:25%) (Figure 3).

Five studies (n = 3064) [8], [29], [34], [38], [40] compared the incidence of SM between *P. vivax* malaria and mixed infection and reported a significantly lower incidence among 5–15 year- age group (summary OR: 0.2, 95% CI: 0.05–0.79, *I* ^2^: 89%) and comparable incidence among infants (summary OR: 0.34, 95% CI: 0.05–2.57, *I* ^2^: 96%) (Figure S2).

### Severe anaemia

**Figure 4:**
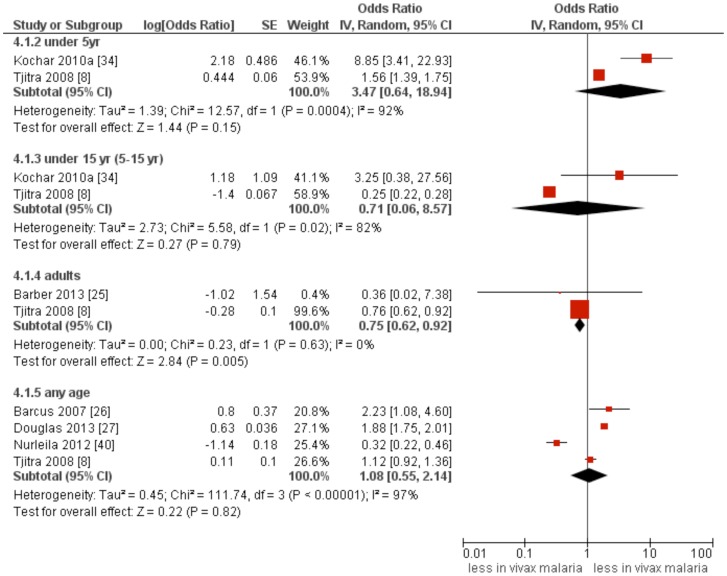
Forest plot showing a comparison of severe anaemia between *P. vivax* and *P. falciparum* infections.

Six studies [8], [25]–[27], [34], [40] which compared the incidences of SA between *P. vivax* infection and *P. falciparum* mono-infection were included in the pooled analysis. A comparable incidence of SA between *P.vivax* infection and *P. falciparum* mono-infection was found in infants (summary OR: 3.47, 95%CI: 0.64–18.94, *I* ^2^: 92%) as well as in the 5–15 year-age group (summary OR: 0.71, 95%CI: 0.06–8.57, *I* ^2^: 82%). Of note, there is substantial between-study heterogeneity. This estimate, however, was significantly lower in adults (summary OR:0.75, 95%CI: 0.62–0.92, *I* ^2^: 0%) (Figure 4). A pooled analysis of 2 studies [8], [34] showed a comparable incidence of SA between *P. vivax* infection and mixed infection (*P. falciparum* and *P. vivax*) in under 5 year-age-group (summary OR: 2.57, 95% CI: 0.12–56.44, *I* ^2^:87%) as well as in the 5–15 year-age group (summary OR: 2.39, 95% CI: 0.94–6.09, *I* ^2^:40%) (Figure S3). Two studies [24], [26] compared incidence of SA between severe and non-severe (uncomplicated) vivax malaria; there was a 6-fold increase in incidence of SA in the group of severe vivax malaria (summary OR: 6.1; 1.92–19.39, *I* ^2^: 0%) (Figure S4).

### Acute respiratory distress in severe vivax malaria

Three studies [8], [25], [26] reported higher incidence of acute respiratory distress (ARD) in *P. vivax* infection compared to *P. falciparum* mono or mixed infection. Two studies on adult participants [8], [25] reported a lower incidence of ARD in *P. vivax* infection compared to *P. falciparum* mono-infection (summary OR: 0.31, 95% CI: 0.17–0.58, *I* ^2^: 0%), whilst 2 studies on children of any age (i.e unspecified age group) [8], [26] showed a comparable incidence of ARD between *P. vivax* and *P. falciparum* infections (summary OR: 1.79, 95% CI: 0.23–14.19, *I* ^2^:61%) (data not shown). Only one study [8] provided data on ARD amongst adult participants, showing a significantly lower incidence of ARD in *P. vivax* infection compared with mixed infection (OR: 0.44, 95% CI: 0.2–0.96).

### Cerebral malaria

One study on adult participants [10] reported a lower incidence of cerebral malaria in *P.vivax* infection than in *P. falciparum* mono-infection (OR: 0.53, 95% CI: 0.32–0.87). However, two studies on children (n = 229) [34], [45] showed a comparable incidence of cerebral malaria between *P. vivax* and *P. falciparum* infections (summary OR: 0.47; 95%CI: 0.12–1.2, *I* ^2^: 0%) (Figure S5).

### Subgroup analysis and sensitivity analysis

**Figure 5:**
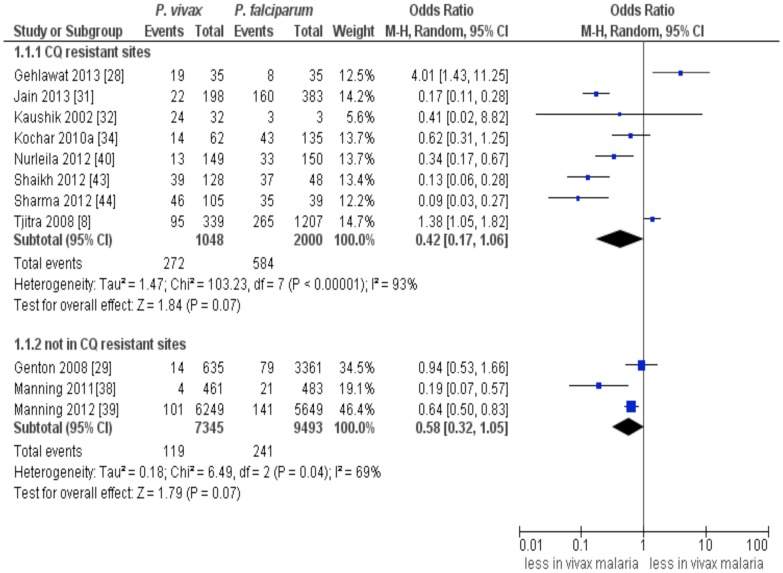
Stratified analysis of severe malaria according to the chloroquine resistant status of the study sites.

Based on available data, we stratified the included studies on SM in the 5–15 year-old children into those which carried out in the study sites where CQ resistance is believed to pay a major role and those with study sites where CQ resistance is not a problem. In both subgroups, a comparable incidence of SM was found between *P. falciparum* infection and *P.vivax infection* (CQ resistant sites: summary OR: 0.42, 95% CI: 0.17–1.06, *I* ^2^:93% and not CQ resistant sites: summary OR: 0.58, 95% CI: 0.32–1.05, *I* ^2^:69%) (Figure 5). Notably, statistical heterogeneity was relatively lower with higher effect estimates and narrower CI among studies in which CQ resistance was not a major problem.

**Figure 6:**
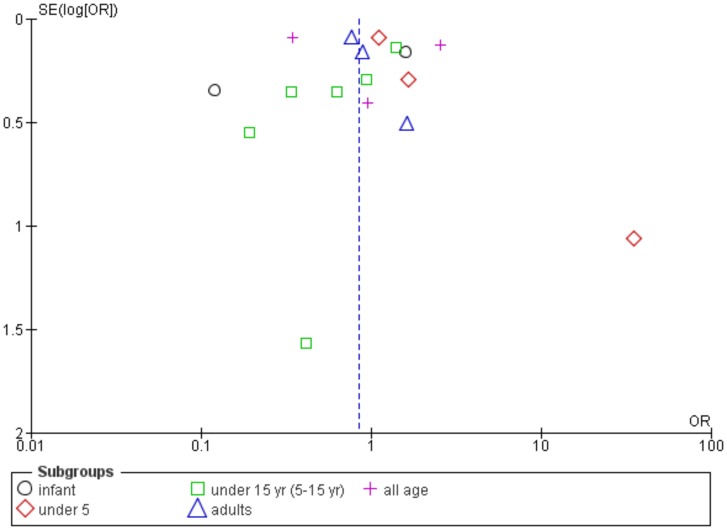
Funnel plot showing publication bias among studies on severe malaria.

A sensitivity analysis including relatively low quality studies showed a significantly lower incidence of SM in *P. vivax* infection than in *P. falciparum* infection amongst the 5–15 year-age group (summary OR: 0.45, 95% CI: 0.25–0.81, *I* ^2^:91%) (Figure S6). Hence, the methodological quality of studies included could have influenced the combined estimates. Compared to *P.falciparum* infection, the 5–15 year-age group with *P.vivax* infection have comparable incidence of SA (summary OR:1.43, 95% CI:0.38–5.35, *I^2^*:79%) (data not shown). An inspection of the funnel plot asymmetry showed a possible publication bias among studies on SM (Figure 6).

## Discussion

Overall, the present analysis identified that the incidence of SM in *P. vivax* infection was considerable, indicating that *P. vivax* infection could be a major cause of SM.

### Seriousness of vivax malaria

Dating back to the era of induced malaria therapy, the fatality rate with the Madagascar strain of *P. vivax*, which is a notoriously virulent strain with relatively good efficacy against neurosyphilis was 10–15% for patients in the United Kingdom [58]. A detail of this evidence is available elsewhere [2]. In fact, vivax malaria is never ever rare and benign [47], [59]. The fact that *P. vivax* has a higher chance of fatality is supported by a study on autopsy case series in Latin America, in which 13 of 17 post-mortem characterization of deceased patients have confirmation of *P. vivax* infections [60].

There are several possible explanations for this observation of severe vivax malaria. A long-lasting liver stage of *P. vivax* allows prolonged periods for the parasite to remain in a host population, even if transmission is interrupted and the primary infection has been treated successfully [61]. It might also be related to recurrent infections resulted from treatment failure of *P. vivax* [8], [29]. Failure to attack the hypnozoite reservoir in patients causes a single infectious bite by a mosquito to result in repeated attacks and opportunities for further transmission [2]. The most frequent malaria related complications reported from *P. vivax* endemic countries are SA and ARD [8], [31], . This pattern was also demonstrated in the current pooled analysis. In settings where malaria is endemic, inadequate therapy and repeated relapses are the primary instruments of severe morbidity and mortality in vivax malaria [2]. Overall, mortality was lower in vivax malaria than in falciparum malaria in certain age groups. Although pathophysiology of *vivax* malaria is not fully understood, the inability of infected red blood cells (RBCs) to adhere to vascular endothelium and the parasite's strict preference for invading reticulocytes could explain the widely accepted ‘nonaggressive course’ of vivax malaria [61].

### Severe anaemia as a presentation of severe vivax malaria

Findings of the current review highlighted that vivax malaria is related to SM, in which SA is a relatively common clinical manifestation. It has been well established that the primary target of human plasmodium species is the RBCs [11]. Mechanisms similar to those operating in the SA of *P. falciparum* could have contributed to the anaemia or there may be different pathways.

*P.vivax* has a very strong predilection for RBCs, particularly reticulocytes, whereas *P. falciparum* has only a moderate predilection [11]. Like in *P. falciparum*, the continued presence of vivax parasites may have been sufficient to infect and destroy most of the new reticulocytes, thereby hindering the timely restoration of the erythrocytes population [62] and this could (partly) lead to extreme anaemia over a period of several months [11]. Moreover, there could have been compounding factors like the impaired immune response of *P. vivax*-infected patients as a result of recrudescence, reinfection, and relapse [63].

### Cerebral malaria and ARD as presentations of severe vivax malaria

The lack of rigidity of infected RBCs in vivax malaria makes the blockage of capillary beds in internal organs unlikely. Thus, the frequency of severe organ-specific clinical manifestations is lower in *P. vivax* infection compared to *P. falciparum* infections [63]. This may be the reason cerebral malaria in adults was less frequently reported in the included primary studies of the present review. A comparable incidence of cerebral malaria between these two infections amongst children found in this review may likely be related to lower immunity level in children than in adults. Published studies had shown that compared to other unusual complications of *P. vivax* infection, cerebral malaria is relatively rare, but when it occurs it is associated with high mortality [63]. Hence, more attention and support should be given to the investigation of *P. vivax* infections in regions such as Indian subcontinent [47]. The presumed pathogenesis of central nervous system malaria is postulated to involve adherence of parasitized RBCs to the cerebral vascular endothelium ultimately impeding the cerebral blood flow [47]. Further production of TNF and subsequent cytokine imbalance are likely to play a role.

Our findings reinforce the finding of individual studies [39], [40], [44] as well as a review on vivax malaria in Brazil [12] that vivax malaria's clinical manifestations such as ARD and death, are comparable in severity to those caused by *P. falciparum*. The lower frequency of ARDS in vivax malaria may be related to the absence of microvascular sequestration of infected RBC [64]–[66], less tissue localization of plasmodium toxin release, resulting in lower degree of microvascular inflammatory response and ischemia-reperfusion injury [65]. Indeed, the pathogenesis of SM is incompletely understood and establishing the causal role of any single mechanism in SM in humans [67] is rather complex. The detailed information on the pathophysiology of vivax malaria is beyond the scope of the current study. Comprehensive information on pathophysiology of vivax malaria pertinent to SA is available elsewhere [11].

### Age-specific severe vivax malaria

Published studies reported that the distribution of SM was age-specific [27], [29]. This is indirectly supported by the current analysis. SA was found to be more frequent in the group of infants infected with *P. vivax*. It has been postulated that a greater risk of SA in early life (infants in this case) could be partly explained by a relatively faster acquisition of immunity in *P. vivax* compared with that in *P. falciparum* [29], [51], [65]. Infancy is a time of rapid physical and cognitive development as well as increased vulnerability to infectious disease. The risk of SA is, therefore, more heavily skewed towards infancy and faster acquisition of immunity [27] in this group of population. A lower incidence of SM in *P.vivax* infection compared with that in mixed infection among the 5–15 year-old children could also be related to the seemingly uniform lower prevalence of vivax malaria in endemic areas. This could partly be attributed to the parasite density.

### Points on diagnosis of severe vivax malaria

The WHO severity criteria formerly only validated for *P. falciparum* infection seems to be applicable to most of the *P. vivax* patients admitted to the intensive care unit (ICU) as at least one of these severity criteria was present in most of the patients admitted [36]. However, definitive criteria for severe disease for *P. vivax* are not validated and the adoption of the WHO thresholds for disease severity for another species needs to be reassessed. For instance, severe disease with *P. falciparum* infection is considered with parasitaemia >200,000/µL, while parasitaemia exceeding 50,000/µL is rare in severe malaria with *P. vivax* [43]. In one study parasitaemia >500/uL was associated with hospitalization in the ICU [36]. Vivax malaria is a potentially life threatening infection despite relatively low-grade parasitaemia in peripheral blood [2]. *P. vivax* infections have lower parasite biomass compared with *P. falciparum* malaria patients of the given age. *P. vivax* has a tendency to achieve and maintain lower density parasitaemia, and the inverse relationship between diagnostic sensitivity and parasitaemia count [61]. This is because the preferential invasion of younger RBCs (reticulocytes) by *P.vivax* lowers the threshold at which it would be considered to be hyperparasitaemic. Another hypothesis is that vivax malaria may be primarily an infection of haemopoetic tissues rather than the vascular sinus. Thus, a biomass of *P. vivax* exterior to the vascular sinus could expand dangerously without detection [2].

### Impact of chloroquine resistance

Geographical areas that reported severe vivax malaria are the same that demonstrated *P. vivax* CQ resistance [60]. The subgroup analysis, based on the CQ sensitivity status in the study sites has indicated that SM was comparable between vivax malaria and falciparum malaria in both sites, regardless of CQ sensitivity pattern. However, relatively lower statistical heterogeneity and slightly higher effect estimates with narrower CI among the studies sites such as East Sepik Province of PNG where CQ resistance is not a major problem or no problem [29]. This implies that the CQ sensitivity pattern reported from the study sites could have influenced the combined estimates. It is important to note the genesis of recommended therapy which constitutes a critical factor for CQ resistance. CQ was commenced as the first line therapy for *P.vivax* in 1946. Although as little as 0.3 gram CQ routinely cured CQ sensitive *P. vivax*, the treatment protocol made no distinction between *P. vivax* and *P. falciparum* for the recommended 1.5 gram total adult dose for treating acute attacks of malaria. This might, in part, explain the relatively late first known appearance of CQ resistant *P. vivax* in 1989 from PNG [68]. Studies have shown that both single nucleotide polymorphism and amplification of *pvmdr1* gene are related to variation in the *in vitro* susceptibility of *P. vivax* similar to *pfmdr1* in *P. falciparum* [68], [69]. When there is a drug resistance trait in a certain area, recurrent infections due to failure to eliminate the parasites early in infection and subsequent relapse from the liver stages [8] resulted in an increased peripheral parasitaemia for a longer time, enhancing haemolysis of RBCs [33] and the subsequent development of SA. Moreover, when compounded by poor immunity, CQ resistant parasites have a greater potential to result in more severe disease, although further studies are needed to confirm this [8]. Confirmation would require evidence of adequate compliance to and absorption of therapy under reliable supervision or, ideally, by determination of the levels of drug (CQ in this case) in the blood. An outstanding research issue related to vivax malaria is addressing the effective alternative therapies for CQ-resistant strains [68].

### Study limitations

Co-infection with other endemic infectious agents could not be ruled out among the participants in the included studies. For instance, a study in Pakistan reported that 12% of patients with vivax malaria had co-existing infection [46]. Hence, there is likely to be an ascertainment bias in the diagnosis of malaria. It is more noticeable in areas of unstable transmission where older adults suffering from chronic conditions such as hypertension, diabetes, or cirrhosis are more prone to develop malarial disease [60]. It was found that age classification was not consistently reported in the included studies. Therefore, the differences in the age groups of participants in the included studies rendered pooled analysis difficult to perform. For instance, the age of participants was classified as 0–5 years including infants [29], while other was classified it separately as infants and 1- <5 year-old [8], [27]. *P. vivax* has a strong predilection for reticulocytes, which are highest in the second postnatal month [70]. Hence, studies in which the analysis of infants and older children are combined might lead to a measurement bias. The present meta-analysis could have been more robust if we had included the relevant literature in non-English languages. Some such literature could have been missed when English-language abstracts were not available. Despite literature search in electronic database using the appropriate search terms, some studies still could have been missed.

Age as a risk factor for SM could have been confounded by the seasonality of malaria in the study settings. This was supported by a prospective study carried out in PNG in which *vivax* malaria patients attending the clinics during wet season had approximately two times greater chance of having SM than those patients during the dry season (OR 1.9,95% CI:1.2–2.9), while it was OR 1.1(95% CI 0.9–1.4) for *P. falciparum* mono-infection and OR 0.8 (95% CI: 0.4–1.9) for *P. falciparum* mixed infections [29]. The year of attendance of patients is likely to impact on caseloads since the implementation of new treatment policy in the study areas could have resulted in fewer cases in post-intervention period of that year [29]. Moreover, manifestations of SM could be influenced by context-dependent factors. The fact that *I^2^* value remains high despite sensitivity analysis implies a *de facto* random distribution of heterogeneity. There may be factors inherent in the included studies such as the level of endemicity, the presence of co-infections, accessibility to effective malaria treatment, and the development of parasite resistance. Inadequate data preclude us from performing stratified analyses based on all these influencing factors.

### Implications

There are important implications for malaria control programmes based on the current findings that PCR confirmed *P. vivax* infections can present as severe disease. The current analysis has documented that the two parasites may occur in equal proportions of SM. The global malaria control strategy and action plan need to readdress the fallacy that vivax malaria is ‘benign’ and not fatal. The vision of no deaths from malaria and a malaria free world requires that vivax malaria should be taken seriously; investments in prevention, diagnosis and treatment strategies to control and eliminate vivax malaria are critically part of achieving this aim.

Because different parasites need to be treated differently, the ability to diagnose the clinical manifestation of vivax malaria at the healthcare provider level must be strengthened. The subsequent appropriate and effective treatment of vivax malaria and monitoring of clinical course and complications can be life-saving. Although the current review has provided evidence of severity of *P. vivax* infection, upcoming well designed prospective studies are needed to substantiate vivax related SM in other epidemiological settings.

## Supporting Information

Figure S1 Forest plot showing a comparison of mortality between *P. vivax* and *P. falciparum* mixed infections.
(PDF)

Figure S2 Forest plot showing a comparative incidence of severe malaria between *P. vivax* and mixed infections.
(PDF)

Figure S3 Forest plot showing a comparative incidence of severe anaemia between *P. vivax* and mixed infections.
(PDF)

Figure S4 Forest plot showing a comparative incidence of severe anaemia between severe vivax and non-severe vivax malaria.
(PDF)

Figure S5 Forest plot showing a comparative incidence of cerebral malaria between *P. vivax* and *P. falciparum* infections.
(PDF)

Figure S6 Sensitivity analysis showing a comparative incidence of severe malaria in the 5–15 year-age group.
(PDF)

Table S1 The characteristic of the included studies.
(RTF)

Table S2 The methodological quality of the included studies.
(RTF)

Checklist S1 PRISMA checklist.
(RTF)
