# Supplementary figures and images for: Is Plasmodium vivax Malaria a Severe Malaria?: A Systematic Review and Meta-Analysis
Source: PLoS Negl Trop Dis. 2014 Aug 14;8(8):e3071. doi: 10.1371/journal.pntd.0003071 (PMC4133404; doi:10.1371/journal.pntd.0003071)

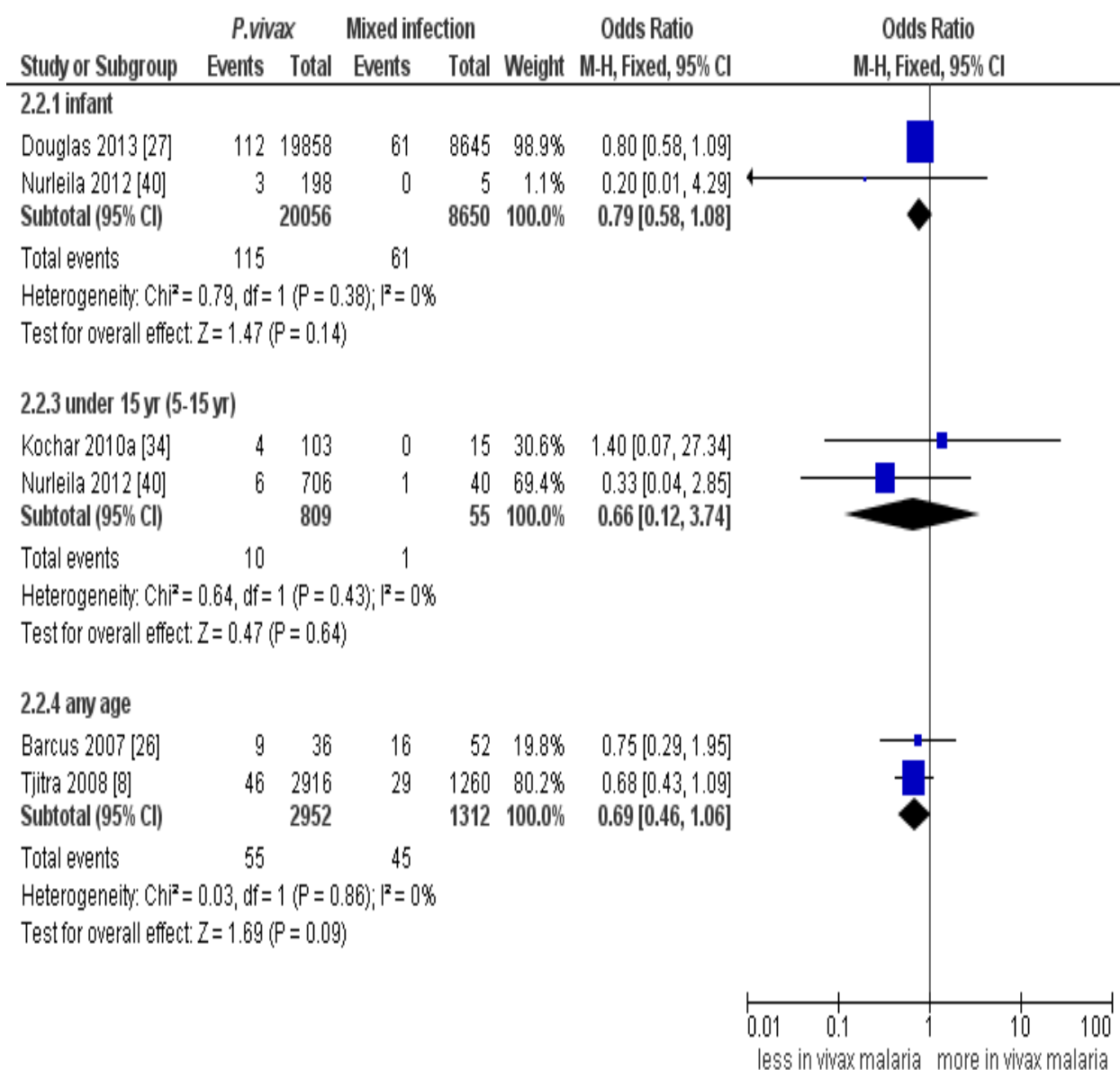

Supplement: Figure S1 — Forest plot showing a comparison of mortality between P. vivax and P. falciparum mixed infections. (PDF) [file pntd.0003071.s001.pdf]

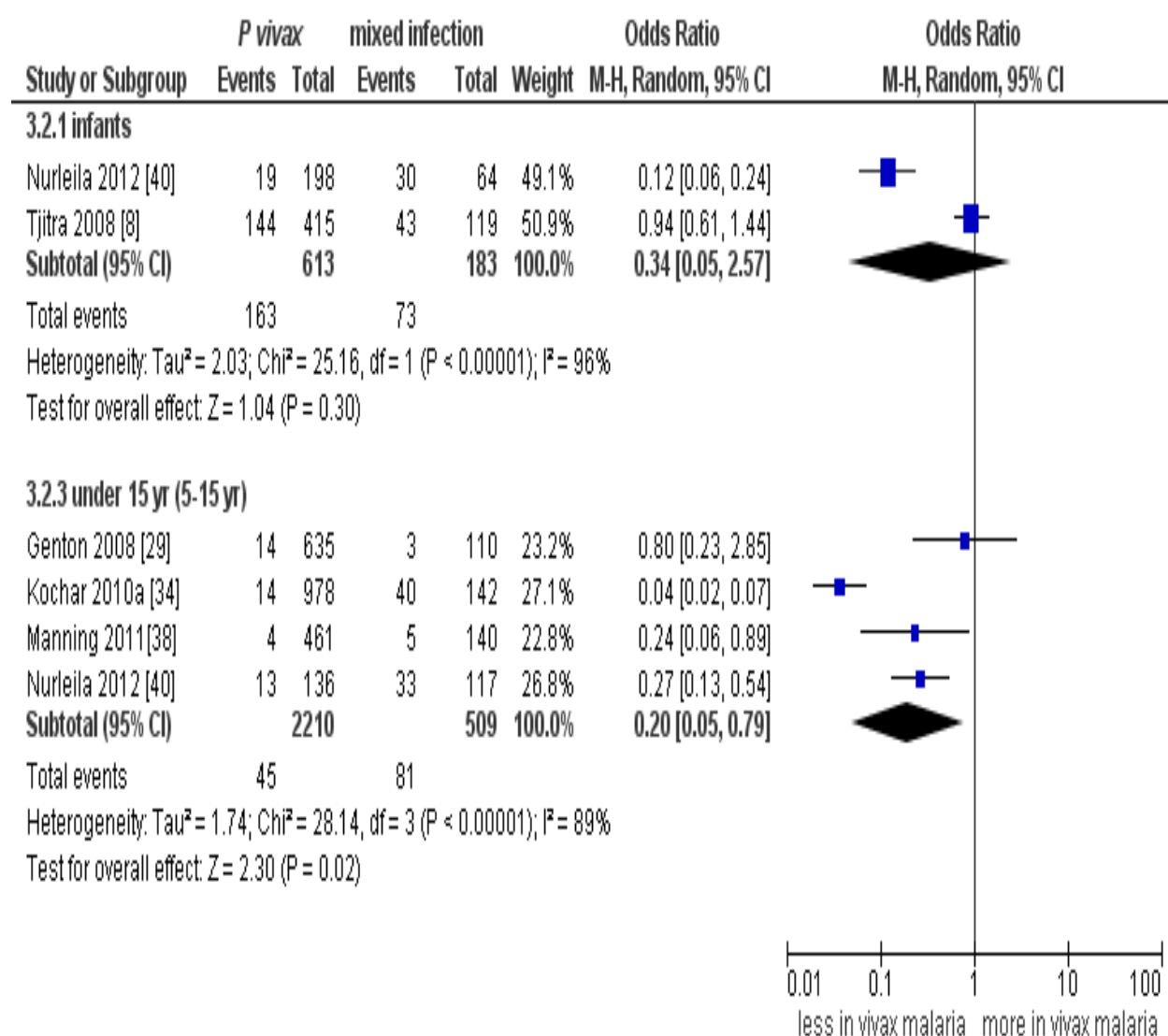

Supplement: Figure S2 — Forest plot showing a comparative incidence of severe malaria between P. vivax and mixed infections. (PDF) [file pntd.0003071.s002.pdf]

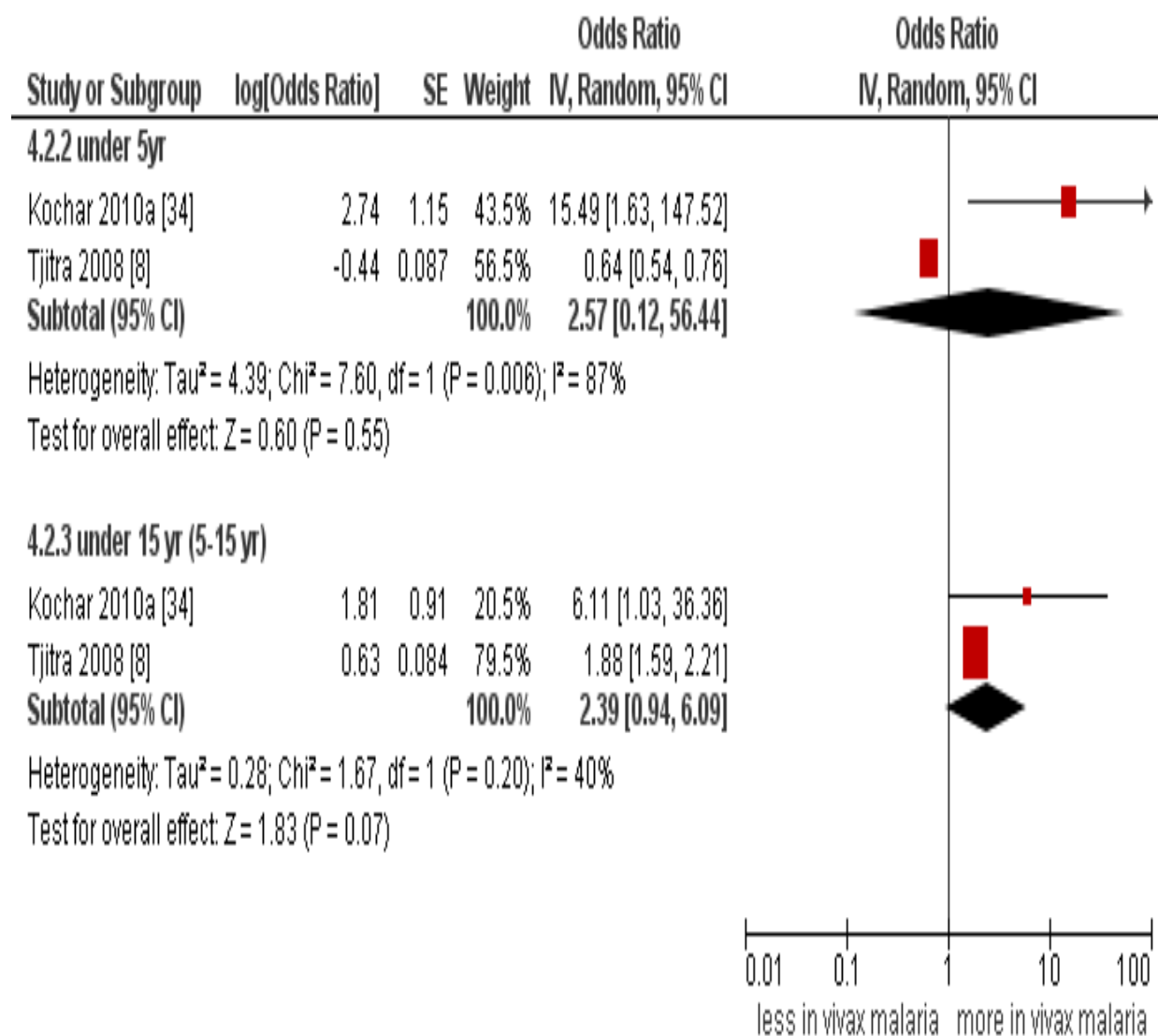

Supplement: Figure S3 — Forest plot showing a comparative incidence of severe anaemia between P. vivax and mixed infections. (PDF) [file pntd.0003071.s003.pdf]

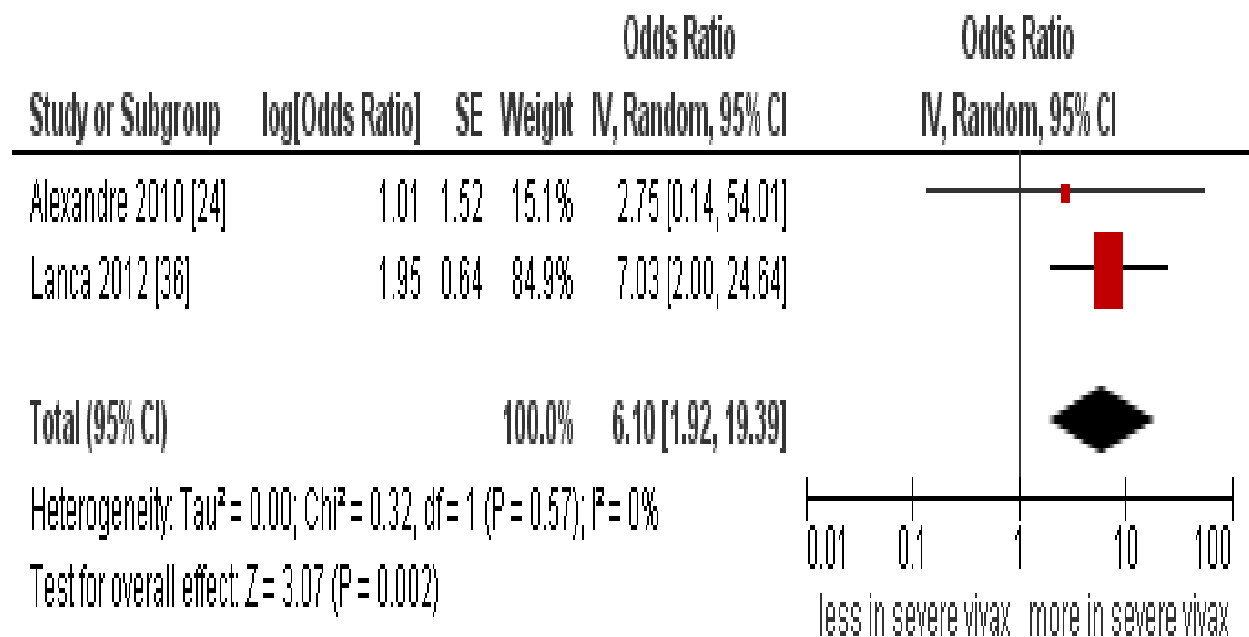

Supplement: Figure S4 — Forest plot showing a comparative incidence of severe anaemia between severe vivax and non-severe vivax malaria. (PDF) [file pntd.0003071.s004.pdf]

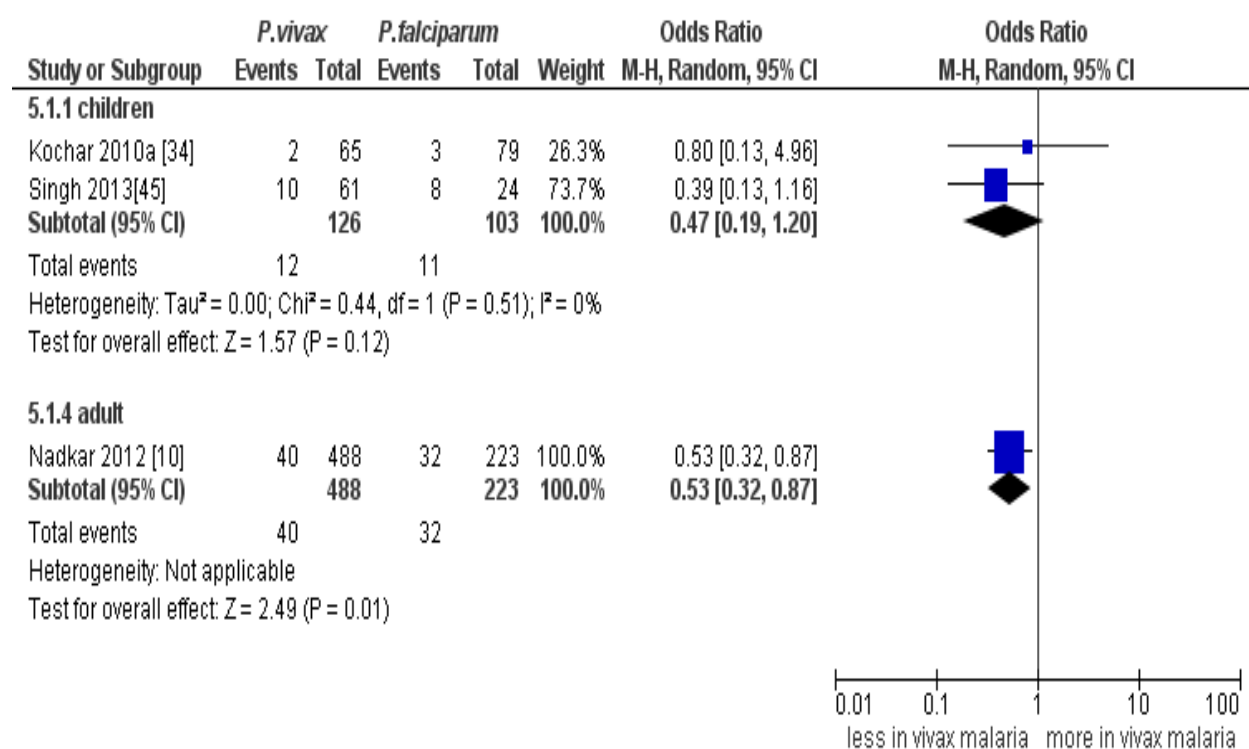

Supplement: Figure S5 — Forest plot showing a comparative incidence of cerebral malaria between P. vivax and P. falciparum infections. (PDF) [file pntd.0003071.s005.pdf]

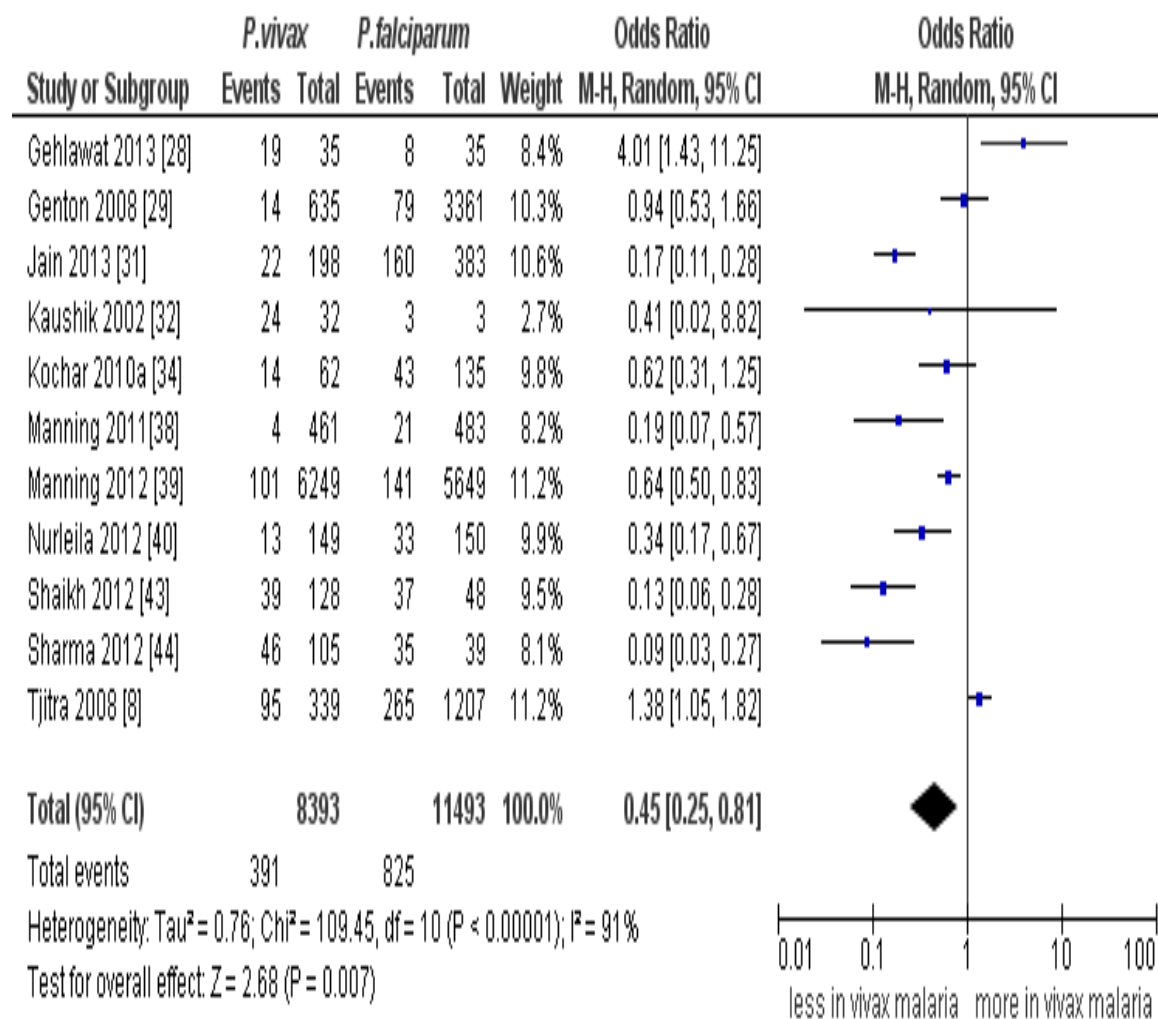

Supplement: Figure S6 — Sensitivity analysis showing a comparative incidence of severe malaria in the 5–15 year-age group. (PDF) [file pntd.0003071.s006.pdf]
