# Supplementary material for: Is Plasmodium vivax Malaria a Severe Malaria?: A Systematic Review and Meta-Analysis
Source: PLoS Negl Trop Dis. 2014 Aug 14;8(8):e3071. doi: 10.1371/journal.pntd.0003071 (PMC4133404; doi:10.1371/journal.pntd.0003071)
Supplement: Table S1 — The characteristic of the included studies. (RTF) [file pntd.0003071.s007.rtf]

Table S1 The characteristic of the included studies

First author [reference no] 	country	study design 	dominant species	transmission	severe vivax/sample size	age in yr, md or mean  (±SD) or ( range)	female %	nPCR2 	main manifestations	remarks	
Tjitra
[8]	Indonesia	prop	Pv	low	16113/60226a;
2917/32171b	<154	65%	not  done	SA, coma, ARD	both inpatients & outpatients;
Infants & 1-<5 years children were separately categorized.	
Nadkar[10]	India	prop	Pv	high	44/488	>21: 88%	28%	not done	TP,CM	mainly adults	
Abdallah, [23]	Sudan	prop, hospitalbased	Pf	unstable	26/139	34.2
(12.6)	42.4%3	not done	hypotension, cerebral malaria	all age	
Alexandre, [24]	Brazil	surveillance	Pv	high	19/219	≤154	42.1%	done	respi failure, SA
	children	
Barber  [25]	Malaysia	prop	Pk	low	43/73	27(17-40)	29%	done	hypotension, jaundice, ARD		
Barcus
[26]	Indonesia	record review	Pf	low	1135/5916	6,mean
30, mean5	<15: 32%;
≥15:68%	done	SA
	9%: pregnant women	
Douglas
[27]	Indonesia	hospital record	Pf	low	544956/28841	NA	87.9%	not done	SA	Participants of all age;
Infants & 1-<5 years children were separately categorized. 	
Gehlawat
[28]	India	prop	Pv	high	18/35	2-5: 38%		not done	SA	children	
Genton, 
[29]	PNG	prop	Pv	perennial	252/7756	<154		done	ARD	Infants were included in the under 5 year-age group	
Haroon, [30]	Pakistan	prop	Pf	NA	110/160	33.2 (± 8.3)	25%3	not done	TP	SM = patients with severe TP	
Jain [31]	India	prop	Pf	high	22/198	9.5, mean	39%	done	CM, 
SA,ARD	mainly children, only 18% (4/22): > 18yr	
Kaushik,
[32]	India	prop, hospital based	Pv	high	35/38	≤ 12	NA	not done	TP, unconscious, convulsion		
Ketema, [33]	Ethiopia	prop, clinic based	Pv	intense	139/478	4.25
(2.95)	42%3	not done	SA,ARD	children	
Kochar [34]	India	prop	Pv	seasonal	65/103	≤ 10	29.4%3	done	multi, SA, TP	children	
Kochr, [335	India	prop,
hospital based	Pv	seasonal	726460	NA	NA	done	TP	adults	
Lanca, [36]	Brazil	record review	Pv	high	64032/52828	≤ 14	29.1% (0.14yr)	not done	ARD, shock,  acidosis	children	
Limaye, [37]	India	retro5	Pv	seasonal	64/338	26		not  known	hypotension, ARD, SA		
Manning [38]	PNG	prop	Pv	high	353/27	3		done	SA	alpha-thalassemia trait: 83.6%1	
Manning [39]	PNG	etiologic	Pv	hyperendemic		38m (24-61m)	49.7%	done	SA	children	
Nurleila [40]	Indonesia	retro, record  		low	3449/1837	<154	80%	not  known	SA,
altered mental state	CQR	
Poespoprodjo [41]	Indonesia	prop,
hospital based	Pf/Pv: 57/43
	low	668/4976	>9-12m; 53.7%		not done	SA	infants	
Rizvi [42]	India	retro,
record	Pv	high	62/172	31.1 (±13.14)	40.3%	not done	SA, CM, ARD,	adults	
Shaikh [43]	Pakistan	retro	Pf	NA	21/64 (2009)
39/128 (2010)	<15		done	convulsion, SA
	children	
Sharma [44]	India	retro, record	Pv	seasonal		56m
(2-168m)	24.8%	not done	SA	children	
Singh, [45]
	India	prop, 	Pv	seasonal	61/85	<18	31.6%	not done	SM, SA, CM	children	
Zubairi,
[46]	Pakistan	retro 	Pv 	NA	111/296	42 ± 18	33%	not done	TP, jaundice, SA	adults	
1:83.6% of children in the study participants; 2: nPCR confirmation of P. vivax with PCR; 3:% of all malaria cases; 4: majority of participants; 5: adult participants; acidosis: metabolic acidosis; Prop: prospective cohort study; retro: retrospective observational/case series study; SA: severe anemia; multi: multiorgan dysfunction; TP: thrombocytopenia; age in year, md: median age in year for vivax mono-infection; a: outpatients; b:inpatients; respi failure: respiratory failure; pregnant women included: pregnant women were included among study participants; CQR: study area is known for Chloroquine resistance; m: months; not done: PCR confirmation was not revealed; RDT: rapid-onsite diagnostic test; Transmission: malaria transmission; Pf: Plasmodium falciparum;  Pk: Plasmodium Knowlesi ; Pv: Plasmodium Vivax; Obs: observational study
