# Supplementary material for: Is Plasmodium vivax Malaria a Severe Malaria?: A Systematic Review and Meta-Analysis
Source: PLoS Negl Trop Dis. 2014 Aug 14;8(8):e3071. doi: 10.1371/journal.pntd.0003071 (PMC4133404; doi:10.1371/journal.pntd.0003071)
Supplement: Table S2 — The methodological quality of the included studies. (RTF) [file pntd.0003071.s008.rtf]

 Table 2 The methodological quality of the included studies
First author (reference no) 	                         selection	comparability	                                 outcome	Total stars	
	representativeness	selection of  non-exposed cohort	ascertainment of exposure	outcome of interested is not at the start of study 	Most important factor	Additional factor	assessment of outcome 
	was follow-up long enough for outcomes to occur
	adequacy of follow up of cohorts
		
Tjitra
[8]	*	*	*	*	*		*	*		7	
Nadkar[10]	*		*		*				*	4	
Abdallah, [23]	*	*	*	*	*		*			6	
Alexandre, [24]	*	*	*		*	*		*	*	7	
Barber  [25]	*	*	*	*	*	*	*	*		8	
Barcus
[26]	*	*	*	*	*		*	*		7	
Douglas
[27]	*	*	*	*	*		*	*		7	
Gehlawat
[28]	*		*	*				*		4	
Genton, 
[29]	*	*	*		*		*	*	*	7	
Haroon, [30]	*			*	*					3	
Jain [31]	*	*	*		*		*	*		6	
Kaushik,
[32]	*		*	*			*	*		5	
Ketema, [33]	*	*	*		*		*		*	6	
Kochar [34]	*	*	*	*	*	*	*			7	
Kochr, [335	*	*	*	*		*			*	6	
Lanca, [36]	*	*	*	*	*	*	*	*		7	
Limaye, [37]	*				*				*	3	
Manning [38]	*	*	*	*	*	*	*		*	8	
Manning [39]	*	*	*	*		*	*			6	
Nurleila [40]	*	*	*	*	*		*	*		7	
Poespoprodjo [41]	*	*	*	*	*					5	
Rizvi [42]	*	*	*					*		4	
Shaikh [43]	*	*		*	*		*			5	
Sharma [44]	*	*	*		*					4	
Singh, 2013 [45]	*		*		*		*		*	5	
Zubairi,
[46]	*			*	*		*	*		5	
