# Supplementary material for: Is Plasmodium vivax Malaria a Severe Malaria?: A Systematic Review and Meta-Analysis
Source: PLoS Negl Trop Dis. 2014 Aug 14;8(8):e3071. doi: 10.1371/journal.pntd.0003071 (PMC4133404; doi:10.1371/journal.pntd.0003071)
Supplement: Checklist S1 — PRISMA checklist. (RTF) [file pntd.0003071.s009.rtf]

Section/topic 	#	Checklist item 	Reported on page # 	
TITLE : Is Plasmodium Vivax Malaria A Severe Malaria?: Systematic Review and  Meta-Analysis		
Title 	1	Identify the report as a systematic review, meta-analysis, or both. 	Title 	
ABSTRACT 		
Structured summary 	2	Provide a structured summary including, as applicable: background; objectives; data sources; study eligibility criteria, participants, and interventions; study appraisal and synthesis methods; results; limitations; conclusions and implications of key findings; systematic review registration number. 	Abstract
	
INTRODUCTION 		
Rationale 	3	Describe the rationale for the review in the context of what is already known. 	Introduction 	
Objectives 	4	Provide an explicit statement of questions being addressed with reference to participants, interventions, comparisons, outcomes, and study design (PICOS). 	Objective
	
METHODS 		
Protocol and registration 	5	Indicate if a review protocol exists, if and where it can be accessed (e.g., Web address), and, if available, provide registration information including registration number. 	PROSPERO 
(CRD 42013006952) 	
Eligibility criteria 	6	Specify study characteristics (e.g., PICOS, length of follow-up) and report characteristics (e.g., years considered, language, publication status) used as criteria for eligibility, giving rationale. 	Study selection provided.  
Participants, outcomes and study design.	
Information sources 	7	Describe all information sources (e.g., databases with dates of coverage, contact with study authors to identify additional studies) in the search and date last searched. 	Study search 
and study selection
	
Search 	8	Present full electronic search strategy for at least one database, including any limits used, such that it could be repeated. 		
Study selection 	9	State the process for selecting studies (i.e., screening, eligibility, included in systematic review, and, if applicable, included in the meta-analysis). 	Study 
selection 
	
Data collection process 	10	Describe method of data extraction from reports (e.g., piloted forms, independently, in duplicate) and any processes for obtaining and confirming data from investigators. 	Data extraction
	
Data items 	11	List and define all variables for which data were sought (e.g., PICOS, funding sources) and any assumptions and simplifications made. 		
Risk of bias in individual studies 	12	Describe methods used for assessing risk of bias of individual studies (including specification of whether this was done at the study or outcome level), and how this information is to be used in any data synthesis. 	Data extraction; The NOS checklist 
	
Summary measures 	13	State the principal summary measures (e.g., risk ratio, difference in means). 	Data analyses
	
Synthesis of results 	14	Describe the methods of handling data and combining results of studies, if done, including measures of consistency (e.g., I2) for each meta-analysis. 		

Page 1 of 2 
Section/topic 	#	Checklist item 	Reported on page # 	
Risk of bias across studies 	15	Specify any assessment of risk of bias that may affect the cumulative evidence (e.g., publication bias, selective reporting within studies). 	A star system indicating the highest star for a study is 9. 	
Additional analyses 	16	Describe methods of additional analyses (e.g., sensitivity or subgroup analyses, meta-regression), if done, indicating which were pre-specified. 	Sensitivity analysis, meta regression
	
RESULTS 		
Study selection 	17	Give numbers of studies screened, assessed for eligibility, and included in the review, with reasons for exclusions at each stage, ideally with a flow diagram. 	Results 
& Figure 1	
Study characteristics 	18	For each study, present characteristics for which data were extracted (e.g., study size, PICOS, follow-up period) and provide the citations. 	Results
& Table S1  	
Risk of bias within studies 	19	Present data on risk of bias of each study and, if available, any outcome level assessment (see item 12). 	Results
& Table S2	
Results of individual studies 	20	For all outcomes considered (benefits or harms), present, for each study: (a) simple summary data for each intervention group (b) effect estimates and confidence intervals, ideally with a forest plot. 	Results
& Fig 2-4, 
Fig S1-S4 
	
Synthesis of results 	21	Present results of each meta-analysis done, including confidence intervals and measures of consistency. 	Summary Odds ratio and its 95% CI 
	
Risk of bias across studies 	22	Present results of any assessment of risk of bias across studies (see Item 15). 	Table S2	
Additional analysis 	23	Give results of additional analyses, if done (e.g., sensitivity or subgroup analyses, meta-regression [see Item 16]). 	Provided sensitivity analysis  Fig S5	
DISCUSSION 		
Summary of evidence 	24	Summarize the main findings including the strength of evidence for each main outcome; consider their relevance to key groups (e.g., healthcare providers, users, and policy makers). 	Discussion
	
Limitations 	25	Discuss limitations at study and outcome level (e.g., risk of bias), and at review-level (e.g., incomplete retrieval of identified research, reporting bias). 	Study limitations
pg#17	
Conclusions 	26	Provide a general interpretation of the results in the context of other evidence, and implications for future research. 	Implications
pg#18	
FUNDING 		
Funding 	27	Describe sources of funding for the systematic review and other support (e.g., supply of data); role of funders for the systematic review. 	 None	

For more information, visit: www.prisma-statement.org. 
Page 2 of 2 
